# Might positron emission tomography actually treat micrometastatic cancer?

**DOI:** 10.3747/co.v16i3.373

**Published:** 2009-05

**Authors:** Mark R. Goldstein, Luca Mascitelli, Francesca Pezzetta

**Affiliations:** Medical Director, Fountain Medical Court, 9410 Fountain Medical Court, Suite A-200, Bonita Springs, Florida 34135, U.S.A, E-mail: markrgoldstein@comcast.net; Comando Brigata Alpina “Julia”, Servizio Sanitario, 8 Via S. Agostino, Udine 33100, Italy, E-mail: lumasci@libero.it; Cardiology Service, Ospedale di Tolmezzo, Tolmezzo 33028, Italy, E-mail: francesca.pezzetta@libero.it

**Keywords:** Micrometastasis, ^18^F-fluorodeoxyglucose, positron emission tomography

The Editor,

Current Oncology

January 21, 2009

We read the interesting pilot study of Dr. Nayot and colleagues on the use of preoperative ^18^F-fluorode-oxyglucose positron emission tomography (fdg-pet) with computed tomography (ct) scanning to detect metastatic nodes in subjects with endometrial cancer^1^. Even though the scanning was not sensitive enough for this purpose, we hypothesize that fdg itself might actually treat occult micrometastatic disease.

A recent retrospective analysis regarding survival in non-small-cell lung cancer (nsclc) demonstrated improved survival among patients with stages iii and iv nsclc in a pet scanning period (1999–2004) as compared with a period before pet scanning (1994–1998)^2^. It was concluded that fdg-pet scanning was, in part, independently associated with improved survival because of more sensitive detection of tumour spread, resulting in stage migration. Interestingly and intriguingly, the survival advantage was seen in patients who experienced fdg-pet scanning regardless of whether they did or did not undergo chemotherapy or standard radiation therapy.

Although the radiation dose from fdg-pet scanning is only a fraction of the standard dose to treat solid tumours^3^, perhaps avid uptake of fdg by metabolically active micrometastatic tumour cells results in highly localized gamma radiation, leading to tumour cell death. Moreover, it is common for lung cancer patients, particularly those with stage iii or iv disease to have circulating micrometastatic tumour cells^4^, which might explain the improved survival among the patients with stages iii and iv nsclc who underwent fdg-pet scanning.

Perhaps fdg should be investigated as a possible treatment modality for micrometastatic tumour burden in various cancers. Furthermore, technologies to determine the presence of micrometastatic disease are available^4^,^5^, possibly enabling treatments to be more specifically targeted.
